# Characterization of callase (β-1,3-d-glucanase) activity during microsporogenesis in the sterile anthers of *Allium sativum* L. and the fertile anthers of *A. atropurpureum*

**DOI:** 10.1007/s00497-012-0184-5

**Published:** 2012-03-23

**Authors:** Krystyna Winiarczyk, Jolanta Jaroszuk-Ściseł, Kamila Kupisz

**Affiliations:** 1 Department of Plant Anatomy and Cytology, University of Maria Curie-Sklodowska, Akademicka St. 19, 20-033 Lublin, Poland; 2 Department of Environmental Microbiology, University of Maria Curie-Sklodowska, Akademicka St. 19, 20-033 Lublin, Poland; 3 Department of Biophysics, University of Maria Curie-Sklodowska, Akademicka St. 19, 20-033 Lublin, Poland

**Keywords:** *Allium sativum*, Callose wall, β-1, 3-D-glucanase-callase, Microsporogenesis

## Abstract

We examined callase activity in anthers of sterile *Allium sativum* (garlic) and fertile *Allium atropurpureum.* In *A. sativum*, a species that produces sterile pollen and propagates only vegetatively, callase was extracted from the thick walls of *A. sativum* microspore tetrads exhibited maximum activity at pH 4.8, and the corresponding in vivo values ranged from 4.5 to 5.0. Once microspores were released, in vitro callase activity peaked at three distinct pH values, reflecting the presence of three callase isoforms. One isoform, which was previously identified in the tetrad stage, displayed maximum activity at pH 4.8, and the remaining two isoforms, which were novel, were most active at pH 6.0 and 7.3. The corresponding in vivo values ranged from pH 4.75 to 6.0. In contrast, in *A. atropurpureum,* a sexually propagating species, three callase isoforms, active at pH 4.8–5.2, 6.1, and 7.3, were identified in samples of microsporangia that had released their microspores. The corresponding in vivo value for this plant was 5.9. The callose wall persists around *A. sativum* meiotic cells, whereas only one callase isoform, with an optimum activity of pH 4.8, is active in the acidic environment of the microsporangium. However, this isoform is degraded when the pH rises to 6.0 and two other callase isoforms, maximally active at pH 6.0 and 7.3, appear. Thus, factors that alter the pH of the microsporangium may indirectly affect the male gametophyte development by modulating the activity of callase and thereby regulating the degradation of the callose wall.

## Introduction

Callose (β-1,3-glucan) is the polysaccharide layer that forms between the plasmalemma and cellulose wall during the growth and development of certain cell types. For instance, callose is present on cell plates during cytokinesis (Hong et al. 2001), in the cells of the abscission layer, and in sieve tubes (Stone and Clarke 1992).

Observable callose deposition occurs within minutes of damage by mechanical, chemical, or ultrasonic treatments and in response to physiological or biotic stress induced by plasmolysis, change in temperature, and microbial infection (Chen and Kim 2009; Stone and Clarke 1992). Callose is thought to play multiple roles in the interaction between a pathogen and its host (Dong et al*.* 2008; Jacobs et al*.* 2003; Nishimura et al*.* 2003; Gomez-Gomez and Boller 2002).

Furthermore, callose occurs in the pollen tube and pollen grain wall (Stone and Clarke 1992). Together with cellulose, callose constitutes the inner lamella of pollen tube walls (Malhó 2006; Vervaeke et al. 2005). The rate of accumulation of callose in pollen tubes can serve as a physiological indicator of compatibility or incompatibility between pollen grains and the stigmatic cell surface (Mollet et al. 2002). In angiosperms, callose plugs are formed at intervals in the growing pollen tubes. Plugs separate the tube into a pollen grain side, which has a large vacuole, and a pollen tube tip, which contains the cytoplasm. Callose plugs maintain a constant amount of cytoplasm, which contains the germ units, in the pollen tube tip (Chebli and Geitmann 2007; Laitiainen et al. 2002). It has been suggested that callose plugs act as mechanical barriers to the reverse flow of plasma from the growing tips to the older parts of pollen tubes (Krabel et al. 1993).

The presence of a callose wall around meiocytes is widely regarded as a prerequisite for meiosis in flowering plants. The wall isolates meiocytes from other sporophytic tissues and, concurrently, prevents them from dehydration in water stress conditions (Li et al. 2010). The callose barrier may serve as a molecular filter that transmits only signals that are indispensable for meiosis into the meiocytes (Dong et al*.* 2005; Rodriguez-Garcia and Majewska-Sawka 2011). Callose is deposited on the external wall of young microsporocytes during the prophase of meiosis, while it forms walls that separate developing microspores after post-meiotic cytokinesis (Waterkeyn and Bienfail 1970). The amount of callose gradually increases during meiosis, but is degraded at the end of microsporogenesis as a result of callase activity (Stieglitz 1977; Chen and Kim 2009). This enzyme can be produced in the gametophytic or sporophytic tissue. Experiments performed by Stieglitz and Stern (1973) suggest that the tapetum, the sporophytic tissue proximal to the tetrads, produces the enzymes necessary for tetrad dissolution. Following degradation of the callosic tetrad walls, the microspores are released into mature pollen grains (Wan et al. 2011; Xie et al. 2010). In the anther locule, free microspores become bicellular pollen grains after asymmetric mitosis and, once they have reached maturity, are released by anther dehiscence.

Callose has an important role in the early deposition of sporopollenin around microspores, the formation of the primary separating wall, and the subsequent formation of a young post-meiotic sporodermal wall (Dong et al*.* 2005; Waterkeyn and Bienfail 1970). The fundamental significance of callose in the formation of functional pollen grains is frequently debated (Fei and Sawhney 1999; Enns et al*.* 2005; Teng et al*.* 2005). Most plants that lack a callose envelope or that have a prematurely disintegrated or abnormally deposited callose envelope give rise to sterile pollen (Abad et al*.* 1995). However, the absence of a callose envelope around meiocytes does not always disturb the normal course of meiosis and pollen development, as has been observed in *Pandanus odoratissimus* (Periasamy and Amalathas 1991), *Lactuca sativa* (lettuce; Curtis et al*.* 1996), *Solanum lycopersicum* (tomato), *Zea mays* (maize), and some transgenic *Nicotiana tabacum* (tobacco) lines (Scott et al*.* 2004).

From early prophase in *Allium sativum* (garlic), a callose wall, which expands gradually during meiosis, appears around the male meiotic cells. The callose envelope reaches maximum size at the stage of the microspore tetrad and persists for over 2 weeks. The microspore tetrads of numerous representative monocots are surrounded by a thick callose wall that persists even when the microspores are surrounded by a new exine layer of sporoderm (Furness and Rudall 1999). The period of wall persistence over 2 weeks observed in *A. sativum* in our study is the longest among all of the genera of *Allium* plants described (Krabel et al. 1993). The structure of the callose wall remains intact, and garlic does not produce any normally viable pollen grains. The persistent callose envelope is likely to cause degeneration of the microspore cytoplasm; however, the destruction of the microspore protoplast may inhibit dissolution of the callose wall. Such “permanent” tetrads have also been described in *Allium schoenoprasum* (Engelke et al*.* 2002) and in male-sterile *Glycine max* (soya bean; Jin et al*.* 1999). It was demonstrated that male sterility is caused by lack of callase activity (Wan et al. 2011), which is responsible for degradation of callose walls. The microspore tetrad fails to disintegrate in microspore (tes) mutants of *Arabidopsis,* in which profound disturbances in cytokinesis were observed (Yang et al*.* 2003).

Our previous long-lasting field observations indicated that degradation of the callose wall surrounding the garlic tetrad was remarkably impeded irrespective of weather conditions (unpublished data). Therefore, in this study, we sought to determine the activity of β-1,3-glucanase, the main enzyme responsible for this process. Furthermore, we made in vivo measurements of pH values in the anther locule, as pH may have a significant effect on callase activity. We employed an original biophysical method of in vivo pH measurements using a specially constructed device.

## Materials and methods

### Plant material

The *Allium sativum* plants used in the study were obtained from the collection at the Maria Curie-Sklodowska University Botanical Garden, Lublin, where, since 1970, they had been reproduced vegetatively from daughter bulbs, the so-called cloves, or by inflorescence bulbils. *Allium atropurpureum* Waldst. & Kit. was chosen as a control species. Species growing on plots in the Botanical Garden or in the greenhouse (one species-*Piper betle*) were used for the comparative study of callase activity:

#### Eudicots:

*Armoracia rusticana* G. Gaertn*.*, *Taraxacum officinale* F. H. Wigg*.*, *Piper betle* L.

#### Monocots:

*Allium sativum* L*.*, *Galanthus nivalis* L*.*, *Miscanthus sinensis* Anderss.

### Microscopy

Floral buds of various sizes were fixed in 1.5 % glutaraldehyde and 1 % formaldehyde in 0.025 M phosphate buffer (pH 7.0) for 1 h at room temperature. The samples were rinsed in the buffer, post-fixed in 1 % osmium tetroxide overnight, dehydrated in a graded ethanol series, infiltrated with propylene oxide, and embedded in LR White. For light microscopy (LM), 1-μm-thick sections were stained with 1 % toluidine blue (in 1 % borax) and examined using the Nikon Optiphot microscope. For transmission electron microscopy (TEM), 60- to 90-nm-thick sections were loaded onto 100-mesh copper grids coated with Formvar (1 % in ethylene dichloride) and stained with uranyl acetate and lead citrate (Reynolds 1963). The sections were viewed using a Tesla BS 500 transmission electron microscope, and images were photographed on Foton TN-12 electron microscope film.

To identify the presence of callose, specific staining with aniline blue and fluorescence microscopy was used. The buds were fixed in a 3:1 mixture of ethanol/glacial acetic acid. Fixed anthers were stained with 50 μg/ml aniline blue in 50 mM KH_2_PO_4_, pH 8.2 (Jensen 1962). The sections were observed under a Nikon Optiphot-2 microscope with a DM 400-nm filter set that detects callose.

### Measurement of pH in the anther locule

Examination of pH in the anther locule was conducted by recording the voltage difference between the H^+^-selective and the reference electrode. An antimony-filled microelectrode, prepared according to a method described previously (Trebacz 1992), was used as the H^+^-selective electrode. The tip diameter of the electrode was 65 μm, and its resistance equaled 52 MΩ. The reference electrode consisted of a silver wire coated with AgCl (Ag/AgCl), placed in a Teflon tube with a porous plug, and filled with 100 mM KCl to yield a salt bridge. The salt bridge and the electrode were immersed in a standard solution to ensure contact between the electrode and the plant. The standard solution contained 1 mM KCl, 0.1 CaCl_2_, and 50 mM sorbitol, pH = 7.0. The microelectrode was inserted into the examined anther locule with an electrical micromanipulator (DC-3 K, Märzhäuser Wetzlar GmbH & Co. KG, Wetzlar, Germany). The active tip of the microelectrode was located extracellularly and sensed pH in the apoplast of the anther locule. Both electrodes were connected to the input of a high-impedance (10^15^ Ω) amplifier Elektrometer Duo 773 (World Precision Instruments, Sarasota, USA). The output signals were digitized and stored on a PC hard drive with BIOWYK software.

Before and after each experiment, the H^+^-selective microelectrode was calibrated with buffer solutions in the pH range of 5–8. Data obtained in the recalibration were the basis of the calculations. The pH values were determined from the calibration curve of the microelectrode.

After measurements were taken, the study anther was transferred into a drop of acetocarmine, and the developmental stages of meiotic cells were identified. The pH value was measured in the anthers of *A. sativum* and in *A. atropurpureum,* the control.

### Specific activity of β-1,3-glucanase (callase) in anthers

Fifty milligrams of anther tissue was pulverized in 500 μl of isotonic extraction phosphate buffer using an agate mortar, and the suspension was centrifuged at 14,000×*g* for 5 min. The supernatant obtained was used for further study. To determine the enzymatic activity of callase, supernatant incubation was performed for 4 h at 37 °C in a mixture consisting of 240 μl of 0.1 M McIllvaine’s buffer, pH 4.8, supplemented with 2 mM β**-**mercaptoethanol, 120 μl of supernatant, 120 μl of solution/suspension of substrate, and carboxymethyl-Curdlan Remazol Brilliant Blue (CM-Curdlan RBB) (Sigma) (Krabel et al*.* 1993).

The reaction was stopped by adding 120 μl of 2 N HCl and cooling to 5 °C. Next, the mixture was centrifuged for 10 min (14,000×*g*), and the supernatant was collected. The optical density of the supernatant was measured at 600 nm. A mixture without the supernatant but with CM-Curdlan-RBB was used as a control. One unit of callase activity (U) was defined as an increase in adsorption at 600 nm per min multiplied by 1000 under the conditions described above.

### Data analysis

All assays were performed in three independent experiments, and the data are expressed as the means ± SD. Significant differences between the means of enzymatic activity and of pH values were determined by analysis of variance (a linear ANOVA model) using Microsoft Excel 97 for Windows (Armitage and Berry 1987). The level of significance was accepted at *P* ≤ 0.05.

## Results

### Localization of callose in the microsporocyte during microsporogenesis

**Fig. 1:**
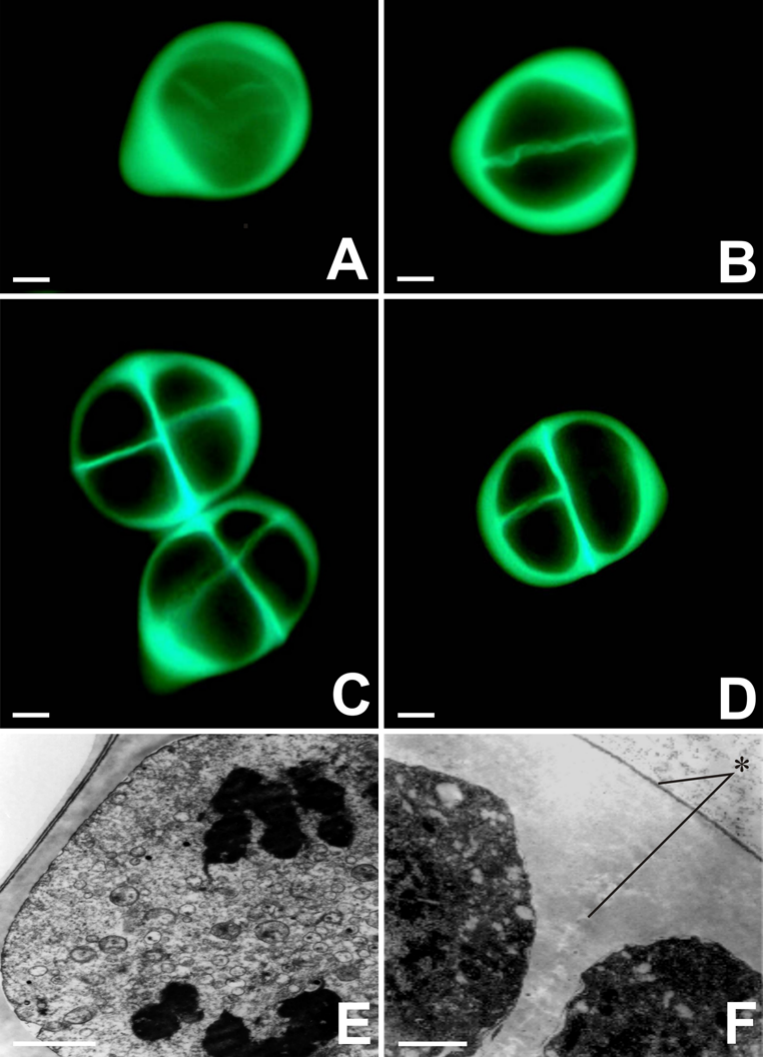
Fluorescence microscopy observation of callose deposition around a meiotic *Allium sativum* cell during the microsporogenesis process. **a** Microsporocyte. **b** Dyad. **c** Tetragonal tetrad configuration. **d** Decussate type. **e** Microsporocyte at anaphase I, surrounded by a *thick* callose wall. **f** TEM image of a fragment of the *thick* external wall around the microspore tetrad (*asterisk*). *Scale bars*: **a**–**d** 2 μm; **e**–**f** 10 μm

We examined the presence of callose in the microsporocyte using fluorescence microscopy and aniline blue staining. In prophase I, the microsporocyte was surrounded by a callose wall that remained intact throughout microsporogenesis (Fig. 1a). During prophase II, each dyad exhibited a thin intersporal callose wall and a considerably thicker external callose wall (Fig. 1b). Complete separation of the four microspores involved the development of a callose wall between them. At the end of meiosis, tetrads of microspores formed with tetragonal (Fig. 1c) and decussate (Fig. 1d) configurations.

The microsporangium of young *A. sativum* anthers was filled with sporogenous tissue, from which microspore mother cells gradually differentiated. Single microspore mother cells had an isodiametric shape, and chromatin condensation occurred in their nuclei. At the pachytene stage of meiosis, deposition of a callose layer on the external wall of male meiotic cells commenced. During anaphase I, a thick layer of callose formed around the microsporocytes (Fig. 1e). After telophase I, simultaneous cytokinesis proceeded, and a thin callose wall was folded in such a manner that it separated the two daughter nuclei. The subsequent stages of meiosis proceeded within one microsporocyte surrounded by a thick layer of callose. The thickness of the internal wall was several times less than that of the external wall. Meiosis in the male meiotic cells was accomplished at the stage of a tetrad of haploid microspores. In the microspore tetrad, a thick external wall and a thin intersporal wall, which represents the primary pectocellulosic wall of the microspore mother cells, were visible (Fig. 1f). The primary pectocellulosic wall of the microspore mother cells disappeared contemporaneously with the thick callosic wall. This stage of male gametophyte development persisted in garlic for over 2 weeks.

Total degradation of the callose wall and release of haploid microspores occurred only during the physiological opening of the bract that surrounds the garlic inflorescence. Initially, the microspores released had a shape corresponding to that of microspores packed in the tetrad, and they remained in this arrangement despite the lack of a callose wall. Next, they adopted an oval shape. At the beginning of the gametophyte generation, all of the microsporangia were filled with mononucleate microspores. Gametogenesis in *A. sativum* was blocked at the microsporocyte stage, and consequently, no functional male gametophytes capable of double fertilization were formed.

### The enzymatic activity of β-1,3-glucanase (callase)

Studies of microsporogenesis in *Allium sativum* demonstrated that the callose wall around the microspore tetrads persisted for about 2 weeks. The first step of the biochemical study was to determine the specific activity of callase in the anthers of *A. sativum* and in plants from the monocot and eudicot clade.

The enzymatic activity of callase from the chosen plant species is summarized in Table 1. Callase isolated from the dicots studied had lower activity than that from the monocot plants analyzed. The level of callase activity in the three monocot species mentioned above was similar and reached over 110 units per milligram of protein. In contrast, callase isolated from the dicotyledonous plants displayed a significantly lower level of enzymatic activity. In *Armoracia rusticana*, callase activity was twofold lower than in *A. sativum*, and in the other species, it was threefold lower.

**Table 1:** β-1,3-glucanase (callase) activity in anthers of different plant species

	Familiae	Species	Activity (U/mg)
Dicot	Brassicaceae	*Armoracia rusticana*	59.73 ± 2.11 B
	Asteraceae	*Taraxacum officinale*	29.14 ± 1.57 C
	Piperaceae	*Piper betle*	30.07 ± 2.49 A
Monocot	Amaryllidaceae	*Allium sativum*	118.81 ± 4.59 A
	Amaryllidaceae	*Galanthus nivalis*	119.95 ± 5.57 A
	Poaceae	*Miscanthus sinensis*	110.76 ± 5.34 A

Mean values in line with different capital letter are significantly different according to the least significant difference (LSD) test (*P* < *0.05*)

**Fig. 2:**
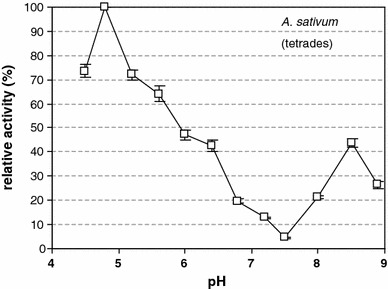
β-1,3-glucanase (callase) activity is dependent on the pH in the *A. sativum* anther during the tetrad stage. A pH of 4.8 corresponded to an activity of 100 %

**Fig. 3:**
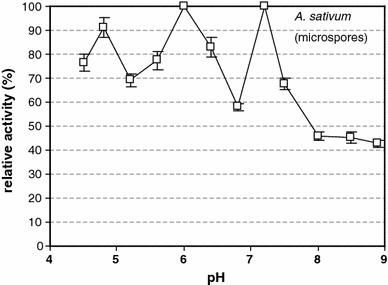
β-1,3-glucanase (callase) activity is dependent on the pH in the *A. sativum* anther during the microspore stage. A pH of 6.0 corresponded to an activity of 100 %

**Fig. 4:**
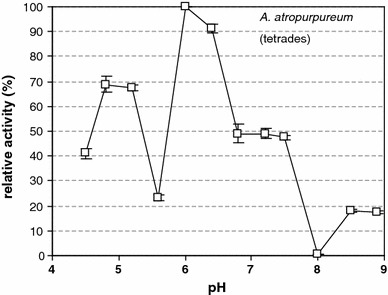
β-1,3-glucanase (callase) activity is dependent on the pH value in the *A. atropurpureum* anther at the tetrad stage. A pH of 6.0 corresponded to an activity of 100 %

Callase activity during microsporogenesis was monitored in *A. sativum* anthers with sterile pollen grain (Figs. 2, 3), whereas *A. atropurpureum* that bore functional male gametophytes was used as control (Fig. 4). In the control species, microspore tetrads were surrounded by a callose wall for about 6 h. pH-dependent callase activity in *A. sativum* was gauged during the tetrad and free microspore stages. In *A. atropurpureum*, however, we analyzed the tetrad stage only, because the interior of the anther collapsed subsequently and the contents of the anther locule spilled out.

In vitro analysis showed that, at the tetrad stage in *A. sativum*, callase activity peaked at a pH of approximately 4.8 (Table 2); there was an inverse relationship between the relative activity of this enzyme and pH both in vitro and in vivo (Table 2; Fig. 2). The analysis of callase activity at the microspore stage indicated three maxima, at pH values of 4.8, 6.0, and 7.3 (Fig. 3), which may indicate the existence of various forms of this enzyme. In the control *A. atropurpureum* plants, at the tetrad stage, the pH value at which callase reached maximal activity was 6.3, but the level of activity reached 70 % of its maximum at pH 4.8 (Fig. 4). In the tetrad stage of *A. sativum*, callase activity peaked only at a pH of 4.8, whereas in the control material (*A.* *atropurpureum*), it peaked at three distinct pH values, that is, 4.8, 6.0, and 7.3.

**Table 2:** β-1,3-glucanase (callase) activity in anthers of *A. sativum* and *A.* *atropurpureum*

	pH		
	4.8	6.0	7.3
*A. sativum* tetrads	274.7 ± 13.54 aA	Not determined	120.4 ± 5.63 bB
*A. sativum* microspores	179.4 ± 5.27 bB	196.8 ± 8.93 aA	190.1 ± 9.00 aA
*A. atropurpureum* tetrads	82.6 ± 4.06 bC	120.1 ± 6.34 aB	52.5 ± 2.63 cC

Mean values in line with different small letter are significantly different according to the least significant difference (LSD) test (*P* < *0.05*)

Mean values in column with different capital letter are significantly different according to the least significant difference (LSD) test (*P* < *0.05*)

### Biophysical investigation of the pH value in the anther locule

The pH value of *A. sativum* anthers filled with the meiotic tissue was investigated in vivo from the youngest stages until the release of microspores. When microsporocytes were present in the anther, the pH in the microsporangium was 4.8. During leptotene, the pH value increased to 5.8, and when the prophase meiocytes were surrounded by a thin callose wall, the pH value reached 6.25. During meiosis, when the callose wall was gradually being formed, the pH value in the anther locule was found to exceed 5.2 (Table 3).

**Table 3:** Values of pH in anthers of *A. sativum* at appropriate stage of meiosis

Stage of meiosis	Archesporial cells	Leptotene	Prophase	Metaphase I and telophase I
pH value	4.80 ± 0.21 d	5.80 ± 0.28 b	6.25 ± 0.14 a	5.20 ± 0.12 c

Mean values in line with different small letter are significantly different according to the least significant difference (LSD) test (*P* < *0.05*)

Measurements made in the *A. atropurpureum* anthers served as a control. Stages measured included tetrads, free microspores, and pollen grains with a formed generative cell. The pH value of the tetrads was 5.9, and that of the mononucleate microspores was 5.5. The pH of anthers containing pollen grains with a formed generative cell was 5.6.

## Discussion

The four haploid microspores of many species of *Allium* are surrounded by a thick callose wall that persists for various periods of time. Wan et al. (2011) showed that the site and timing of callose degradation are important; a delay in the release of microspores affects the subsequent development of pollen, resulting in male sterility. The long-term persistence of the callose wall surrounding the microspore tetrads is confirmed by observations of the ‘Piemonte’ garlic clone (Gori 1983) and *Allium tuberosum* (Bhandari et al*.* 1981). In this study, we established that the callose wall of *A. sativum* persisted for more than 2 weeks, which is the longest period of any *Allium* plant hitherto described. It is possible that the long-lasting callose wall caused the microspore cytoplasm to degenerate. Another possibility is that the tapetum, which is responsible for production of enzymes taking part in male gametophyte development, was not fully functional and did not synthesize callase, and as a consequence, the callose wall was not degraded (Fei and Sawhney 1999).

Investigations of sterile lines of flowering plants showed correlations between particular developmental stages of meiotic cells and the activity of some enzymes involved in the processes of meiosis and gametogenesis (Wu and Murry 1985). The most important of these enzymes is callase (β-1,3-glucan), which is responsible for the proper development and maturation of fertile pollen (Enns et al*.* 2005). As early as 1977, Stieglitz reported a considerable increase in the callase activity in *Lilium* from the stage of tetrads until the end of gametogenesis. The post-meiotic release of microspores from the common callose wall is strictly conditioned by the activity of callase (Mamun et al. 2005; McCormick 1993). This enzyme is transported from the tapetum to the anther loculus as early as during the initial meiotic stages (Fei and Sawhney 1999). Since the tapetum is responsible for the production of callase, any disturbances in the functioning of tapetum are reflected in the amount of callase or in its activity. Callase release into the anthers of petunia and lily is strictly controlled by the β-Glu gene, and distortions in the expression of this gene lead to disturbances in the digestion of callose walls, which is probably the primary cause of the appearance of cytoplasmic male-sterile lines in petunia, sorghum, and soybean (Leubner-Metzger and Meins 1999), and in *Arabidopsis* (Fei and Sawhney 1999).

Callase retains its enzymatic activity for approximately 48 h after it has been produced in the anther; it is most active during the second meiotic division and in microspores at the tetrad stage (Stieglitz and Stern 1973). According to early studies conducted by Mepham and Lane (1969), callase retains its activity for only 24 h, while a study by Stieglitz and Stern (1973) suggested that the enzyme remains active throughout microsporogenesis, but that the level of activity varies. The highest level of callase enzymatic activity was noted during microspore release. Changes in the pattern of enzymatic activity of callase (Majewska-Sawka and Rodriguez-Garcia 1999), which has been shown to exhibit functional activity in tapetum cells (Pacini 1990), is one mechanism that disturbs the process by which microspores and pollen grains form; these disturbances often lead to male cytoplasmic sterility.

The specific activity of callase largely depends on the pH in the microsporangium. The ambient pH determines which isoform of callase is maximally active and can be a key factor in the development of a functional male gametophyte (Frankel et al*.* 1969; Izhar and Frankel 1971). In our study, callase extracted from microsporangia containing tetrads surrounded by thick callose walls exhibited maximum activity at pH 4.8. The pH values measured in vivo in the same microsporangia fluctuated from 4.5 to 5.0. Similar measurements were taken on a control plant, *A. atropureum*, during degradation of the callose envelope around the tetrads. In this case, three callase isoforms, active at pH 4.8–5.2, 6.1, and 7.3, were revealed. In contrast, the pH value measured in vivo was 5.9. Studies of the changes in the activity of this enzyme were carried out on *Lilium* (Stieglitz 1977), *Arabidopsis* (Dong et al*.* 2005), and transgenic tobacco (Worrall et al*.* 1992). It was demonstrated that the pH in the anther locule of male-sterile *Petunia hybrida* lines was higher, ranging from 6.8 to 7.0 during meiosis, and that callase was inactive under these conditions. Under these conditions, microspore tetrads remained enclosed in the intact callose wall for an extended period of time. In fertile genotypes, however, the pH values at the tetrad stage declined to 5.9–6.2 (Izhar and Frankel 1971). In our study, in vivo measurements showed that pH values varied from 4.75 to 6.0 in *A. sativum*, when the microsporangia contained microspores released from the callose envelope. In contrast, the in vitro examination of enzymatic callase activity in these stages revealed three pH values at which the three isoforms of callase reached maximum activity. One isoform displayed maximum activity at pH 4.8 (it had previously been identified in the tetrad stage); additionally, two novel isoforms appeared that were active at pH 6.0 and 7.3. These enzymatic activity parameters for callase are very similar to those found for the control plants, *A. atropurpureum,* during the earlier tetrad stage. In light of recently published data (Chen and Kim 2009; Wan et al. 2011; Vervaeke et al*.* 2005) and our own results, we propose that there is great variety in the characteristics of callase isoforms, not only for specific plant species, but also for the particular meiotic stages. Such vast differences in the specific activity of the enzyme may have physiological consequences. In the case of *A. sativum,* a reduction in callase activity may inhibit the disintegration of the callose wall around the microspore tetrad.

The inhibited dissolution of the callose wall may also be related to the presence of callase inhibitors in the plant or to other modulating/modifying factors, such as cellulase activity (Sexton et al*.* 1990; Dong et al*.* 2005). This hypothesis is justified, because the special wall surrounding the microspore tetrad is composed of callose and unesterified and methyl-esterified pectins (Majewska-Sawka and Rodriguez-Garcia 1999). It may be assumed that callase is not the only callose wall-dissolving enzyme in *A. sativum*, because, despite its high activity, this process proceeds very slowly. Our results obtained for *A. sativum* show that the callose wall persists around meiotic cells, while in the microsporangium, an acidic environment of pH 5.0 or less is maintained, and only one callase isoform is active, with optimum activity at pH 4.8. However, degradation of the callose wall occurs only when the pH rises to 6.0 and the two other callase isoforms that are active at pH 6.0 and 7.3 appear. These findings are in agreement with the results obtained using the control plants, *A. atropurpureum*, where degradation of the callose wall took place at similar pH values in the microsporangium and three isoforms of callase were present.

This study shows that factors that affect the pH in the microsporangium may be indirectly associated with infertility of garlic due to distortions in callase activity and degradation of the callose wall. Our novel method of directly measuring pH inside microsporangium seems to be very useful in monitoring the activity of enzymes in the microsporangium during microsporogenesis.

## Conclusion

The main cause of sterility in this species is the lack of a functional male gametophyte, as there is no gametogenesis in the microspores. This dysfunction is probably due to the lack of degradation of the callose wall around the microspore tetrad, which leads to microspore abortion.

Investigations of the enzymatic activity of callase demonstrated the existence of three isoforms of this enzyme that are active at specific/different pH values. Therefore, we assume that at least one of the callase isoforms can lead to degradation of the callose wall. Since the callose wall is no longer degraded and the callose envelope persists for more than 2 weeks, gametogenesis does not occur and no functional male gametophyte is formed, and instead, degeneration of the microspore cytoplasm is observed. This results in microspore abortion and the inability of this species to undergo generative reproduction. It has been shown that callase isoforms are enzymatically active; therefore, a reduction or inhibition of enzyme activity may be caused by the presence of some inhibitors. A number of such inhibitors have been isolated and characterized from legume seeds, cereals, and tubers (Shivaraj and Pattabiraman 1981; Elemo et al. 2011).
